# RMP promotes epithelial-mesenchymal transition through NF-κB/CSN2/Snail pathway in hepatocellular carcinoma

**DOI:** 10.18632/oncotarget.16177

**Published:** 2017-03-14

**Authors:** Wei Zhou, Qi Wang, Yi Xu, Jingting Jiang, Jingchun Guo, Huijun Yu, Wenxiang Wei

**Affiliations:** ^1^ Department of Cell Biology, Institute of Bioengineering, School of Medicine, Soochow University, Suzhou 215123, China; ^2^ Department of Tumor Biotherapy, Third Affiliated Hospital of Soochow University, Changzhou 213003, China; ^3^ State Key Laboratory of Medical Neurobiology, Fudan University, Shanghai 200032, China

**Keywords:** RMP, EMT, HCC, NF-κB, metastasis

## Abstract

Epithelial-mesenchymal transition (EMT) is a significant risk factor for metastasis in hepatocellular carcinoma (HCC) patients and with poor prognosis. In this study, we demonstrate the key role of RPB5-mediating protein (RMP) in EMT of HCC cells and the mechanism by which RMP promote EMT. RMP increases migration, invasion, and the progress of EMT of HCC cells, which facilitates the accumulation of Snail, a transcriptional repressor involved in EMT initiation. NF-κB is activated by RMP, which directly promotes the expression of COP9 signalosome 2 (CSN2) to repress the degradation of Snail. Pulmonary metastases mouse model demonstrates that RMP induces metastasis *in vivo*. Immunohistochemical analysis of human HCC tissues confirms the correlation of RMP with the expression of E-cadherin, p65, CSN2 and Snail *in vivo*. Collectively, these findings indicate that RMP promotes EMT and HCC metastasis through NF-κB/CSN2/Snail pathway. These results suggest that RMP and p65 may serve as potential candidates of the targets in the treatment of metastatic HCC.

## INTRODUCTION

Hepatocellular carcinoma (HCC) is the fifth and ninth leading cause of cancer-related death in male and female worldwide [1]. The high recurrence and low five-year survival rate of HCC is mainly due to the intrahepatic and extrahepatic metastases [2]. Intrahepatic metastasis is frequently associated with epithelial mesenchymal transition (EMT) [3]. Metastasis of tumor cells is a complex process. It has been demonstrated that EMT is the first key step in metastatic cascade and is critical during the early events of tumor cell metastasis for endowing cells with stronger motility and invasiveness [4]. The mechanism underlying the malignant phenotype of HCC is largely unknown. Enhancing our knowledge of the molecular mechanisms of EMT will help us to increase the overall prognosis of patients with HCC by targeting metastatic therapy.

RPB5-mediating protein (RMP), also assigned as Unconventional prefoldin RPB5 interactor (URI), was firstly identified to bind to RPB5, one of the subunits of RNA polymerases [5]. Thereafter, the interaction of RMP/URI with chromatin through RNA polymerase II was found mostly targeting in the transcription of nutrient-sensitive genes [5, 6]. Recently, studies uncovered that RMP/URI played positive roles in carcinogenesis [7]. In HCC cells, RMP/URI significantly promoted proliferation and diminished radiation-induced apoptosis. Interaction between RMP/URI and HBx in HCC cells may responsible for Hepatitis B virus-associated hepatocarcinogenesis[8, 9].

The rising protein level of RMP/URI has been testified in human ovarian cancer [7], cervical cancer [10], multiple myeloma [11] and portal vein tumor thrombus (PVTT) which raised from HCC [12]. Prompted by these observations, the molecular mechanism of RMP/URI in EMT and metastasis is still elusive.

In this study, we investigate the key role of RMP in EMT of HCC cells and the mechanism by which RMP mediates EMT. We demonstrate that RMP promotes migration, invasion, and the progress of EMT of HCC cells *in vitro* and HCC metastasis *in vivo*. Further studies reveal that RMP activates NF-κB/CSN2/Snail pathway, instead of Akt/GSK-3β, to repress Snail degradation and induce EMT and metastasis.

## RESULTS

### RMP promotes migration and invasion of HCC *in vitro*

**Figure 1:**
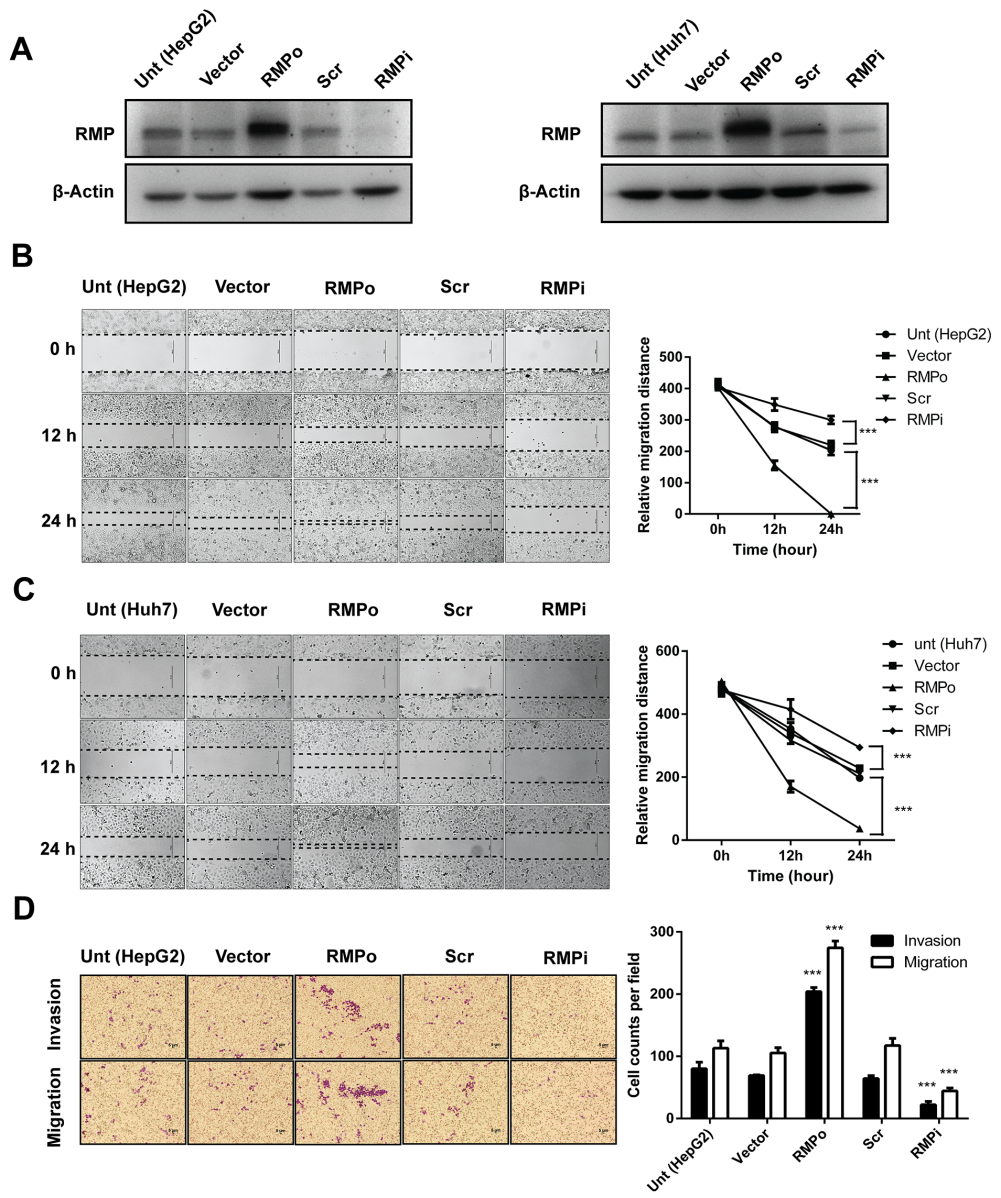

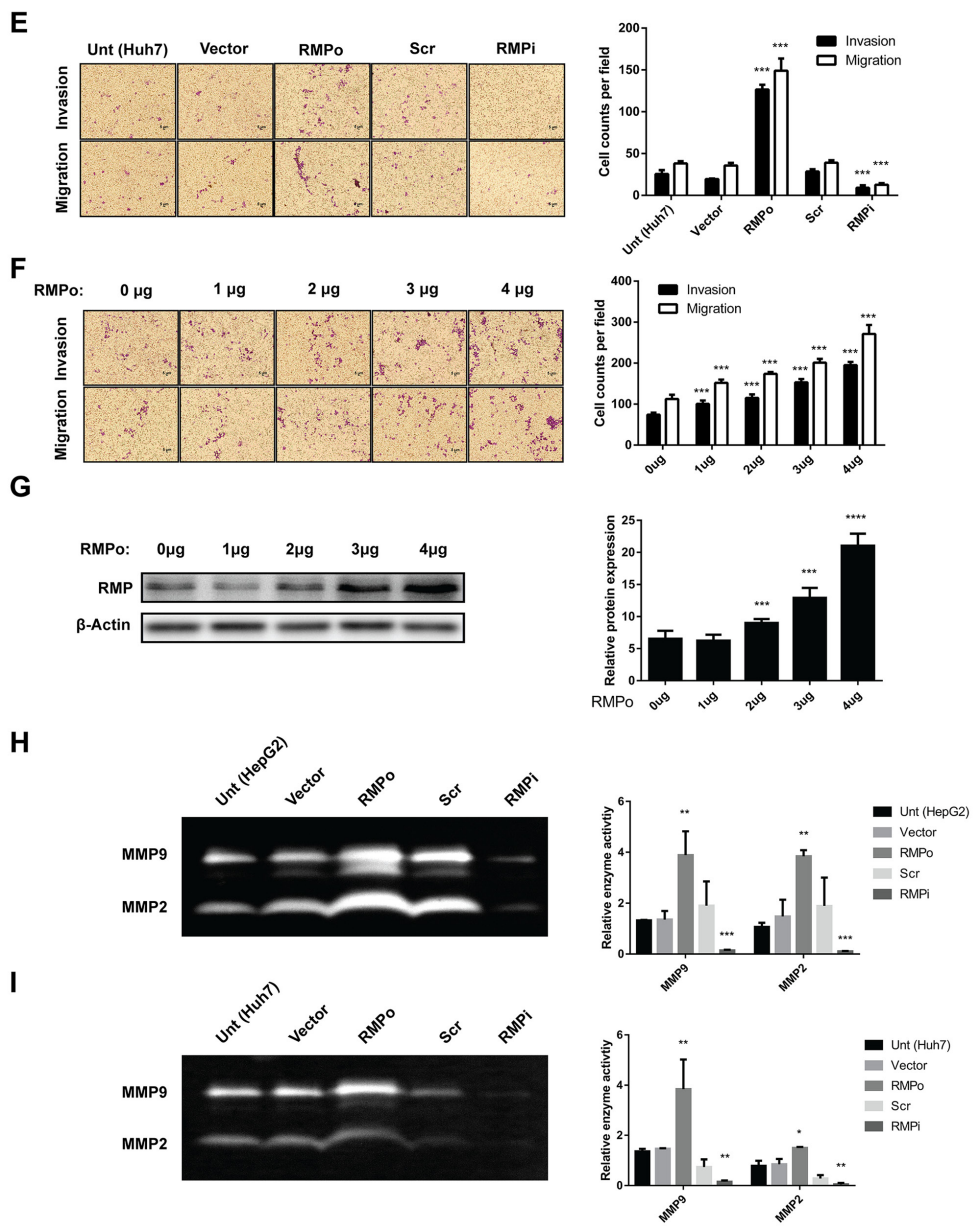
RMP promotes migration and invasion of HCC cells *in vitro* HepG2 (left panel) and Huh7 (right panel) cells were transfected with empty vector (Vector), pCDNA3.1-RMP overexpression vector (RMPo), Scramble shRNA sequence (Scr) and pGPU6-RMP shRNA vector (RMPi) respectively. **(A)** The expression of RMP in transfected HepG2 (right panel) and Huh7 (left panel) cells was examined by western blot, β-Actin was shown as loading control. **(B-C)** Wound-healing assay was carried out to determine the migration of HepG2 (B) or Huh7 (C) cells transfected with indicated plasmids. The width of the wound was measured in 0, 12 and 24 hrs after wounding. Scale bar, 50 μm. Width of wound was scored and depicted graphically (right panel). **(D-E)** Migration (without matrigel) and invasion (with matrigel) of HepG2 (D) or Huh7. (E) cells transfected with indicated plasmid were detected by transwell system. Cells were stained with crystal violet after 24 hrs incubation. Scale bar, 5μm. Number of invaded cells were scored and depicted graphically (right panel). **(F)** HepG2 cells were transfected with 0, 1, 2, 3, 4 μg of pCDNA3.1-RMP overexpression vector (RMPo) as indicated. Migration (without matrigel) and invasion (with matrigel) were detected by transwell system. Scale bar, 5μm. The number of invaded cells was scored and depicted graphically (right panel). **(G)** HepG2 cells were transfected with increasing amount of pCDNA3.1-RMP overexpression vector (RMPo) as indicated in (F) and were harvested 48 hrs later. Cell lysates were subjected to western blot analysis with antibody against RMP. **(H-I)** HepG2 (H) or Huh7 (I) cells were transfected with indicated plasmids. Gelatinase activities of cell culture supernatant of each cell were measured by zymographic assay, and the band in 96 kDa and 72 kDa represents the activity of MMP9 and MMP2 respectively. Gelatinase activities were scored and are depicted graphically (right panel). The results are expressed as the mean ±SD of three independent experiments, each measurement was made in triplicates (*, P < 0.05, **, P < 0.01, ***, P < 0.001, ****, P < 0.0001, independent Student t test).

Aberrant overexpression of RMP in various malignant tumors is correlated with a poor prognosis. To determine whether RMP induces EMT in HCC, we constructed plasmid vehicles which contain interfering sequence and whole coding sequence (CDS) of RMP. The cell lines of RMP overexpression (RMPo) and RMP depletion cell (RMPi) were generated with HepG2 and Huh7 (Figure 1A) cells by transfection. As shown in (Figure 1A and Supplementary Figure 3), the protein level of RMP was elevated in RMPo cells from both HepG2 and Huh7. While the expression of RMP was significantly decreased in RMPi cells compared with cells transfected with empty vector (Vector) or scramble shRNA sequence (Scr).

Then we examined the effect of RMP on the migration of HCC cells. Wound healing assay was applied to test migration. The movement of cells across the scratched area of the cell monolayer indicates the migration is a process independent of proliferation. RMPo cells of both HepG2 (Figure 1B) and Huh7 (Figure 1C) migrated more rapidly compared with the control cells and covered the wound in 24 hrs. The ability of cells to migrate was significantly reduced when RMP was depleted.

To confirm these results, we examined the cells with an alternative method of migration and invasion. Transwell system was applied as described in the Material and Methods. As shown in (Figure 1D), higher invasion and migration ability were observed in RMPo cells than untransfected cells or cells transfected with vector alone. In contrast, the migration of RMPi cells was significantly slower than cells transfected with empty vector or Scr (Figure 1D). To further confirm, Huh7 cells were also subjected to the analysis. The results showed that RMP is also necessary for the migration and invasion of Huh7 (Figure 1E). Next, we confirmed the effect of RMP on migration and invasion of HCC cells in a dose dependence manner. HepG2 cells were transfected with increasing amounts of RMP expression vector (Figure 1F), which was confirmed by the western blot analysis (Figure 1G). Interestingly, the increasing amount of RMP expression resulted in increasing promotion of migration and invasion (Figure 1F). As invasive phenotypes were always linked to the activation of matrix metalloproteinase MMPs (MMPs) family, we also examined the expression of MMP2 and MMP9 in these cell lines [13, 14]. The results showed both MMP2 andMMP9 increased in RMPo cells, and significantly decreased in the RMPi cells of HepG2 (Figure 1H), which was also observed in Huh7 cells (Figure 1I). These results confirmed that increasing RMP expression is responsible for the highly migration and invasion of HCC cells, which provide strong support for our hypothesis that RMP was the key regulator in metastasis of HCC.

### RMP promotes the progress of EMT *in vitro*

**Figure 2:**
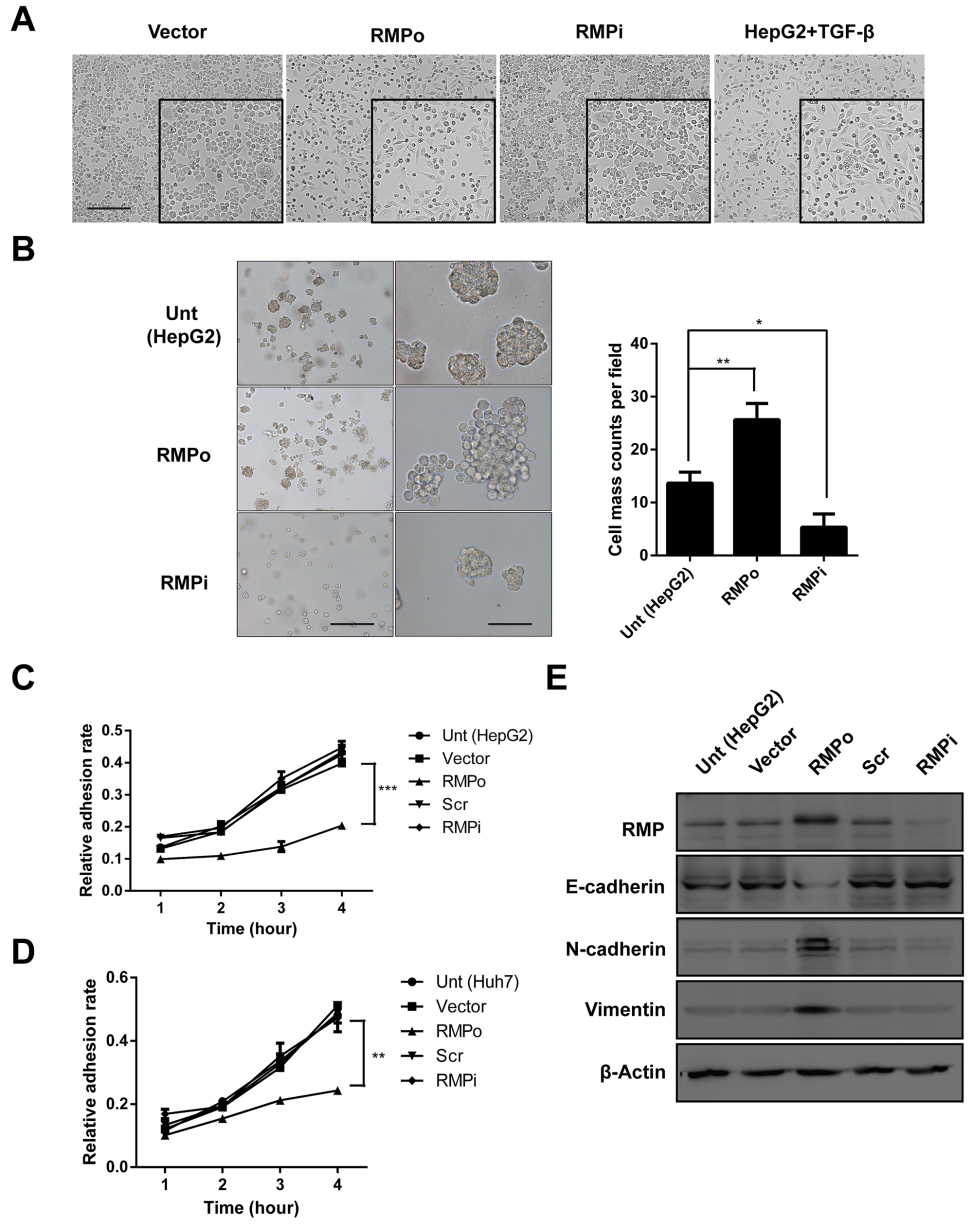
RMP promotes the progression of EMT *in vitro* HepG2 cells were transfected with pCDNA3.1-RMP overexpression vector (RMPo) or pGPU6-RMP shRNA vector (RMPi). **(A)** HepG2 cells transfected with indicated plasmid was followed by cultured for 72 h. Wild type HepG2 cells treated with TGF-β (10 ng/ml) for 72 hrs and applied as a positive control of EMT. The morphology of cells was examined under microscope. Scale bar, outside 20 μm; inside 5 μm. **(B)** HepG2 cells transfected with indicated plasmid were subjected to sphere forming assay. After culture in suspension for 14 days, sphere number were counted in 3 random fields of microscope and depicted graphically (right panel). Scale bar, left panel 50 μm; right panel 5 μm. **(C-D)** Cell adhesion to fibronectin was examined in HepG2 (C) and Huh7 (D) cells transfected with empty vector (Vector), pCDNA3.1-RMP overexpression vector (RMPo), scramble shRNA sequence (Scr) and pGPU6-RMP shRNA vector (RMPi) respectively. After 1 to 4 hrs culture, non-adhesion cell were washed by PBS. Adhesion cells were calculated and depicted graphically. **(E)** The expression of RMP, E-cadherin, N-cadherin, Vimentin and Tubulin were analyzed in HepG2 transfected with indicated plasmid by western blot. β-Actin was shown as loading control. The results are expressed as the mean ±SD of three independent experiments, each measurement was made in triplicates (*, P < 0.05, **, P < 0.01, ***, P < 0.001, independent Student t test).

Epithelial-mesenchymal transition (EMT) is well known to occur at the tissue development, wound healing process and the invasive front of metastatic cancers [15–17]. Acquisition of the mesenchymal phenotype has been associated with invasiveness *in vivo* and *in vitro*. In some cases, E-cadherin-negative cells showed higher levels of cell migration and invasion [4, 18]. We examined the effect of RMP on the EMT of HCC cells. The RMPo cells grow vigorously compared with cells transfected with vector alone or cells depleted of RMP. This morphology of RMPo cells were similar to the phenotype of HepG2 cells treated with TGF-β [19, 20], which serve as positive control of EMT (Figure 2A). Next we examined HCC cells by sphere-forming assay. The results demonstrated that HepG2 cells with RMP overexpression displayed highly capability of soft-agar colony formation. In contrast, HepG2 cells with RMP depletion (RMPi) showed decreased capability of colony formation (Figure 2B). To evaluate the effect of RMP on the expression of stem cell marker, CD44, CD90, CD133 and EpCAM were examined by quantitative RT-PCR. And the results showed that CD44 and EpCAM were significantly increased in RMPo cell (Supplementary Figure 1A). As cell adhesion is an important element for EMT we examined the effect of RMP on the adhesion of HCC cells. The HepG2 cell was examined for the cell adhesion after transfection. The results showed that the overexpression of RMP (RMPo) significantly reduced the adhesion rate of HepG2 cells (Figure 2C), which is a common feature of malignant tumor. Interestingly, depletion of RMP did not affect the adhesion ability of HepG2 cells, which is known as a benign feature (Figure 2C). To confirm this conclusion, an alternative HCC cell line, Huh7 were also examined for the adhesion assay. As shown in (Figure 2D), the adhesion of Huh7 cells overexpressing RMP were significantly reduced, which was consistent with those observation in HepG2. These results showed that RMP plays a negative role in the adhesion of HCC cells.

Cadherin and Vimentin are the most critical markers of epithelial and mesenchymal cells, which facilitate EMT and tumorigenesis. We examined the effect of RMP on the expression of these EMT factors. As shown in (Figure 2E and Supplementary Figure 4), overexpression of RMP reduced the expression of E-cadherin and increased mesenchymal maker N-cadherin and Vimentin. These experiments strongly suggest RMP is an important regulator of EMT factors.

The expression of RMP in HepG2 and Huh7 cells was generally low, which is the reason why these cells were sensitive to overexpression of RMP rather than depletion of RMP. This explains that RMPo significantly changed the expression of EMT markets (Figure 2E) and reduced adhesion of HCC cells (Figure 2C and 2D). However these HCC cells were insensitive to RMP depletion so that no differences was observed in the expression of EMT markers (Figure 2E) and cell adhesion (Figure 2C and 2D) between these HCC cells and the RMPi cells.

### RMP-mediated EMT is dependent on stabilization of Snail in HCC cells

**Figure 3:**
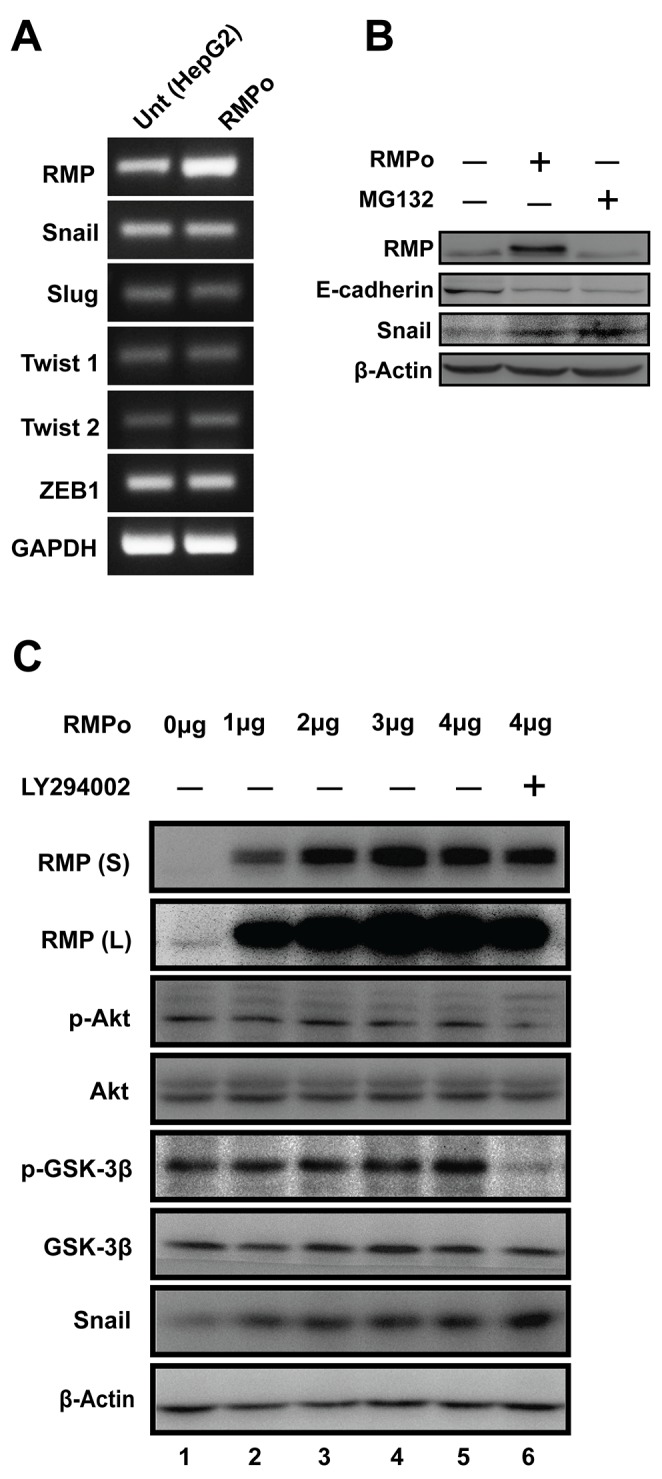
RMP-mediated EMT is dependent on stabilization of Snail in HCC cells **(A)** The mRNA expression of RMP, Snail, Slug, Twist1, Twist2, ZEB1 and GAPDH in HepG2 cells transfected with pCDNA3.1-RMP overexpression vector (RMPo) was measured by RT-PCR. GAPDH was shown as loading control. **(B)** HepG2 cells were untransfected or transfected with pCDNA3.1-RMP overexpression vector (RMPo) followed by treatment with or without proteasome inhibitor MG132 for 12 hrs. Expression of RMP, E-cadherin, Snail and β-Actin was examined by western blot. β-Actin was shown as loading control. **(C)** HepG2 cells were transfected with 0, 1, 2, 3, 4 μg pCDNA3.1-RMP overexpression vector (RMPo) followed by treatment with or without 50 μM Akt inhibitor LY294002 for 24 hr. Proteins expression were analyzed by western blot analysis with antibodies as indicated. RMP(S) and RMP (L) represent the blots with short or long time exposure, respectively. β-Actin was shown as a loading control. The results are expressed as the mean ±SD in three independent experiments.

Most of EMT processes are influenced by intra- or extra-cellular signal induced gene transcript variation. As E-cadherin was down-regulated in RMPo cells, we expect several transcriptional factors to be involved in RMP-induced EMT. As shown in (Figure 3A), no change was observed in mRNA expression of Snail, Slug, Twist1, Twist2 or ZEB1 in RMP overexpression cells (RMPo). To further confirm these results, quantitative RT-PCR was performed, which was consistent with the results above (Supplementary Figure 2). As Snail is reported to be regulated in the level of protein stability, western blot analysis was performed. A proteasome inhibitor, MG132 was applied to evaluate stabilization of Snail protein. As show in (Figure 3B and Supplementary Figure 5A), Snail is remarkably elevated in HepG2 cells pre-treated with MG132 or overexpressed RMP respectively. Moreover, we also observed the epithelial marker of E-cadherin was dramatically decreased by RMP overexpression, which was similar to the effect of MG132 treatment (Figure 3B), indicating RMP and MG132 both promote the accumulation of Snail, and eventually lead to the degradation of E-cadherin. These results suggest that RMP functions in stabilizing Snail, which is essential for EMT transition.

Akt is frequently activated in human epithelial cancer, and Akt/GSK-3β/Snail signaling pathway was involved in EMT process. Therefore, we first examined whether the stabilization of Snail by RMP is mediated by regulation of Akt or GSK-3β pathway. Increasing dosage of RMPo vector were transfected into HepG2 cells (Figure 3C and Supplementary Figure 5B). The p-Akt (Ser-473) and p-GSK-3β (Ser-9) remain unchanged regardless of the increasing expression of RMP (Figure 3C), while Snail was found to be upregulated by the increasing RMP level (Figure 3C), suggesting that RMP-mediated stabilization of Snail was not through the phosphorylation of Akt and GSK-3β. To further address this possibility, RMPo cells were treated with PI3K/Akt inhibitor LY294002 to repress activation of Akt. After 24 hrs incubation, phosphorylation of Akt was well blocked and results in basal state of GSK-3β (Figure 3C, lane 6). Interestingly, we observed increased Snail in cells additional treated with LY29002 compared to RMP overexpression alone (Figure 3C), suggesting that GSK-3β remain inactivated in HCC cells and the function of GSK-3β and RMP in the regulation of Snail may be paralleled. Collectively, RMP mediated stabilization may not be through Akt pathway in HCC cells.

### NF-κB, but not Akt is required for RMP-mediated stabilization of Snail

**Figure 4:**
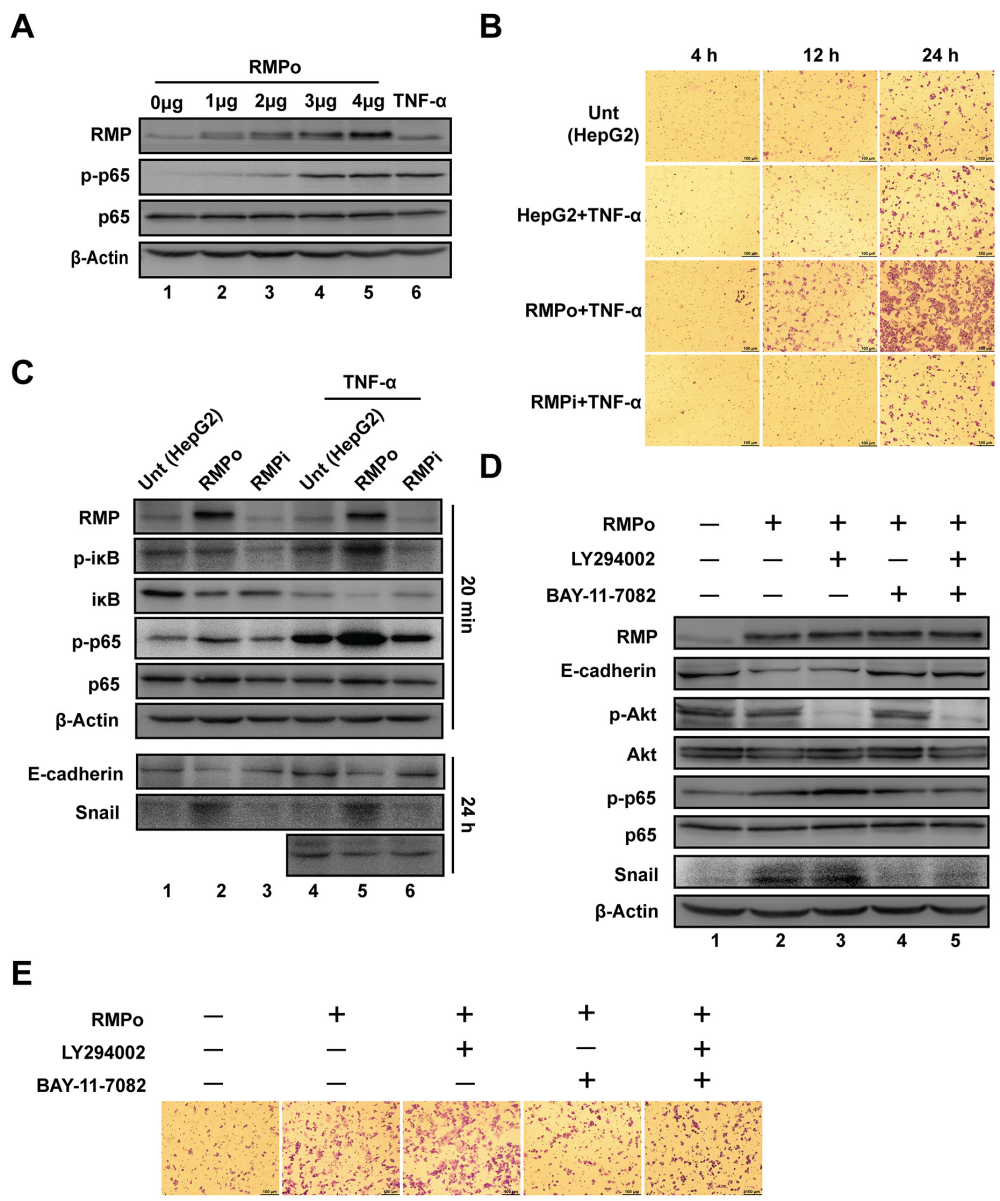
NF-κB, but not Akt is required for RMP-mediated stabilization of Snail **(A)** HepG2 cells were transfected with 0, 1, 2, 3, 4 μg of pCDNA3.1-RMP overexpression vector (RMPo). Wild type HepG2 cells with 8 hrs TNF-α treatment were applied as a positive control of NF-κB activation. Then expression of RMP, p-p65, p65 and β-Actin were examined by western blot. β-Actin was shown as a loading control. **(B)** HepG2 cells were transfected with indicated plasmids and then treated with or without 10 ng/ml TNF-α for 24 hrs. Cell invasion was detected in 4, 12 or 24 hrs respectively. Scale bar, 100μm. **(C)** HepG2 cells were transfected with various plasmids and then treated with or without 10 ng/ml TNF-α for 20 min or 24 hrs as indicated, respectively. The protein expression was analyzed by western blot with the indicated antibodies. **(D)** HepG2 cells transfected with pCDNA3.1-RMP overexpression vector (RMPo), followed by treatment with or without 50 μM LY294002 or 8 μM BAY-11-7082 for 24 hrs and protein expression was analyzed by western blot with antibodies indicated, β-Actin was shown as loading control. **(E)** HepG2 cells transfected with or without pCDNA3.1-RMP overexpression vector (RMPo) were treated with or without 50 μM LY294002 or 8 μM BAY-11-7082, and then subjected to transwell assay. The experiments were performed in three independent experiments.

NF-κB has previously been found to facilitate EMT and inhibit the degradation of Snail [21]. Dose dependent assay was carried out to address the possibility that NF-κB is involved in RMP induced Snail stabilization and EMT. As shown in (Figure 4A and Supplementary Figure 6A), the phosphorylation of p65 (Ser-536) was enhanced by the increasing expression of RMP, similar to the effect of TNF-α, which was served as a positive control to induce phosphorylation of p65.

Next, we examined the cooperative effects of RMP and TNF-α on the invasion of HCC cells. The invasion was increased in RMPo cells after treatment with TNF-α (Figure 4B). Surprisingly, TNF-α treatment did not increase the invasion of HepG2 and RMPi cells (Figure 4B). These results demonstrate that, RMP is a key regulator of activity of p65, which plays an important role in the downstream activation in NF-κB signal transduction.

p65 is associated with its inhibitor iκB to form hemo- or hetero- dimers among Rel family transcription factors. We further examined if RMP mediated Snail stabilization by NF-κB pathway. HepG2, RMPo and RMPi cells were treated with TNF-α for 20min to activate NF-κB. As (Figure 4C and Supplementary Figure 6B) show, iκB was degraded in RMPo cells as the degradation of iκB is closely linked to the phosphorylation of its Ser-32. Specifically, the total expression of iκB was decreased by RMP overexpression and further decreased by TNF-α treatment (Figure 4C, lane 2 & 4-6), phosphorylation of iκB (Ser-32) was significantly increased by TNF-α in RMPo cells (Figure 4C, lane 5), which is consistent with the degradation of iκB. However, the phosphorylated iκB in RMPi cells after TNF-α treatment remained unchanged, means RMP is necessary for iκB phosphorylation (Figure 4C). Meanwhile, we also examined the expression of Snail and E-cadherin in cells treated with or without TNF-α for 24 hrs. We also evaluated the expression of Snail and E-cadherin in cells treated with or without TNF-α. Snail was only increased in RMP overexpression cells regardless of TNF-α treatment. This effect was also identified by the decreasing of E-cadherin in RMPo cells (Figure 4C).

To confirm the critical role of NF-κB in RMP-mediated Snail stabilization, RMPo cells were treated with inhibitors of Akt (LY294002), NF-κB (BAY-11-7082) separately or combined. As (Figure 4D and Supplementary Figure 6C) show, the phosphorylation of Akt and p65 was well blocked by their inhibitors (Figure 4D line 3 and 4). The expression of Snail was increased in the cells overexpressing RMP (Figure 4D, lane 2). Inhibition of Akt with LY294002 did not affect the expression of Snail (Figure 4D, lane 3). However inhibition of NF-κB with BAY-11-7082 significantly suppressed the expression of Snail (Figure 4D, lane 4). Treatment with both LY294002 and BAY-11-7082 reduced the expression of Snail to a level similar as treatment with BAY-11-7082 alone (Figure 4D, lane 5). Meanwhile, E-cadherin was significantly decreased by the increasing expression of Snail which suppressed the transcription of E-cadherin (Figure 4D). These results suggest that NF-κB, but not the Akt, is required for the RMP-mediated stabilization of Snail which in turn drives EMT in HCC cells.

We further confirm the effect of LY294002 and BAY-11-7082 on the invasion of HCC cells by transwell assay. Consistent with the results above, the invasion was inhibited by BAY-11-7082, but not LY294002 in RMPo cells (Figure 4E). Collectively, these results clearly indicate that the RMP is responsible for the activation of NF-κB pathway, which prevents degradation of Snail and thus enhances cell invasion and EMT.

### RMP up-regulated CSN2 expression through nuclear translocation of NF-κB

**Figure 5:**
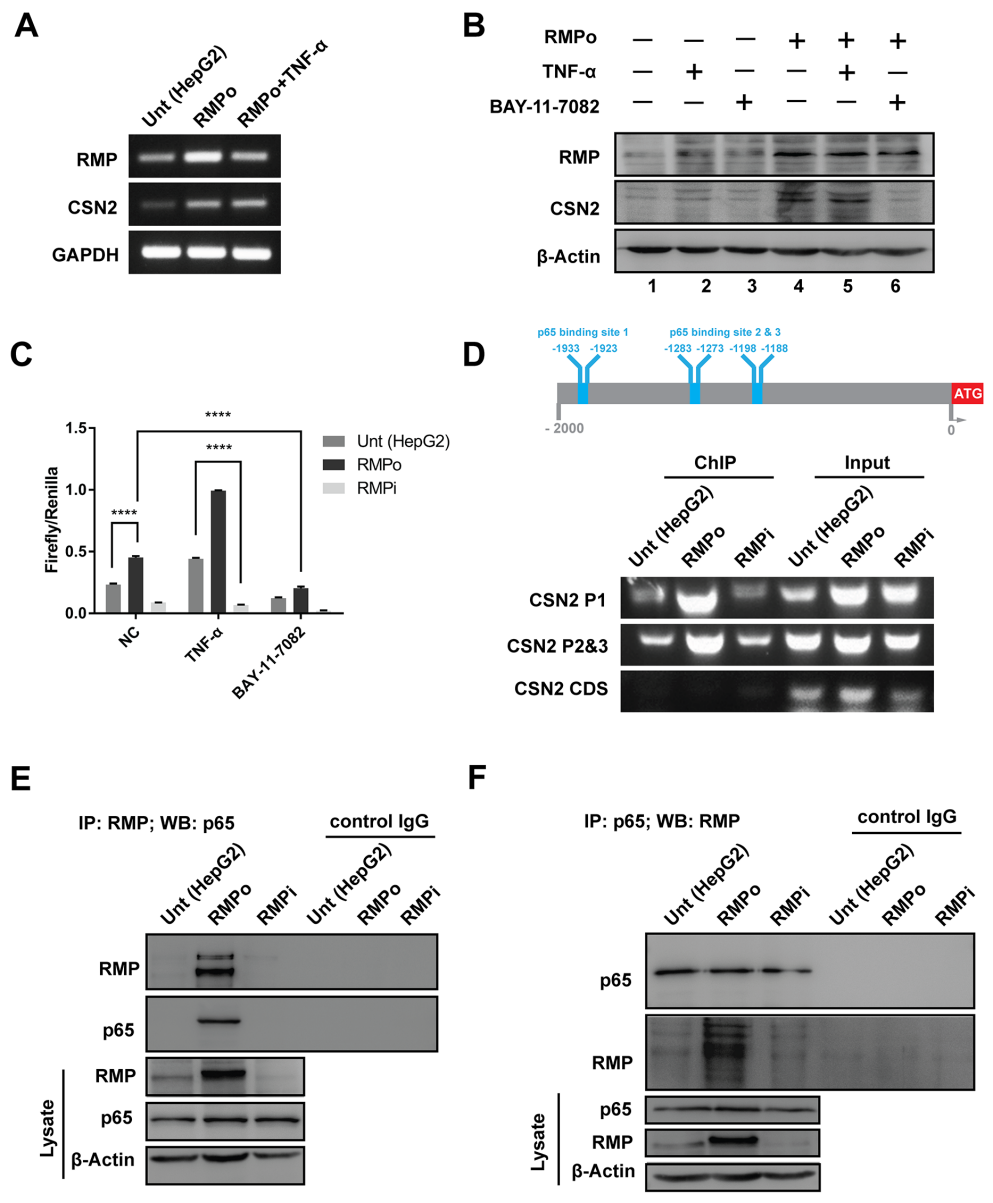

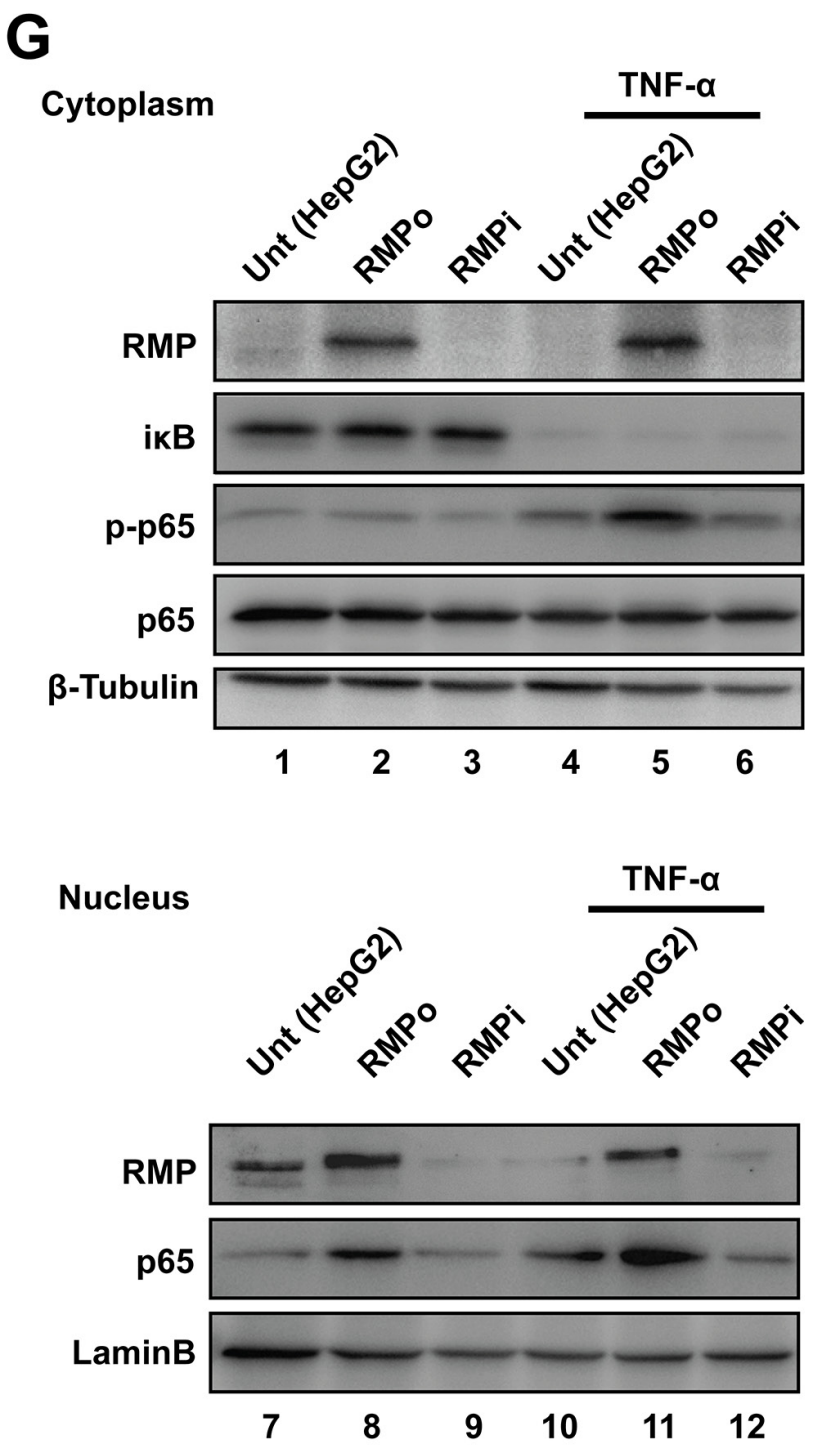
RMP up-regulated CSN2 expression through nuclear translocation of NF-κB **(A)** HepG2 cells transfected with pCDNA3.1-RMP overexpression vector (RMPo) for 24 hrs and followed by culturing with or without 10 ng/ml TNF-α for additional 24 hrs. Then the mRNA expression of RMP, CSN2 and GAPDH were analyzed by RT-PCR. GAPDH was shown as loading control. **(B)** HepG2 cells transfected with pCDNA3.1-RMP overexpression vector (RMPo) for 48 hrs and cultured with or without 10 ng/ml TNF-α and 8 μM BAY-11-7802 for additional 24 hrs. Protein expression of RMP, CSN2 and β-Actin were analyzed by western blot, and β-Actin was shown as loading control. **(C)** HepG2 cells were co-transfected with pCDNA3.1-RMP overexpression vector (RMPo), pGL3-CSN2-promoter vector and pGL4.74 vector for 48 hrs, then cultured with or without 10 ng/ml TNF-α and 8 μM BAY-11-7802 for additional 24 hrs. Promoter activity of CSN2 was detected by dual-luciferase assay. **(D)** Schematic of CSN2 promoter, which contains 3 NF-κB binding sites. CSN2 promoter region 1 (P1) containing p65 binding site 1 and CSN2 promoter region 2 & 3 (P2&3) containing p65 binding site 2 and 3 (upper panel). HepG2 cells were transfected with pCDNA3.1-RMP overexpression vector (RMPo) or pGPU6-RMP shRNA vector (RMPi) for 24 hrs, followed by treatment with or without 10 ng/ml TNF-α for additional 8 hrs. Chromatin immunoprecipitation (ChIP) was carried out with antibodies as indicated. Promoter fragment of CSN2 was immunoprecipitated by p65 antibody. The coding region of CSN2 was used as negative control (bottom). **(E-F)** HepG2 cells were stably transfected with pCDNA3.1-RMP overexpression vector (RMPo) or pGPU6-RMP shRNA vector (RMPi). Cell lysates were extracted and immunoprecipitation was performed with antibodies against RMP (E) or p65 (F). The immunoprecipitates were fractionated in 10% SDS-PAGE and subjected to western blot analysis with antibodies against RMP and p65 as indicated. Preimmune IgG was applied as control of immunoprecipitation (Control IgG). **(G)** Cytoplasm and nucleus fractions of HepG2 cells were stably transfected with indicated plasmid or treated with 10 ng/ml TNF-α for 20 min, then subjected to western blot. Lamin B and β-tubulin were used as the endogenous control from nucleus and cytoplasm, respectively. The results are expressed as the mean ±SD in three independent experiments (*, P < 0.05, **, P < 0.01, ***, P < 0.001, ****, P < 0.0001, independent Student t test).

COP9 signalosome 2 (CSN2) is the most conserved subunit of COP9 signalosome, which is linked to E3 ubiquitin ligases in the degradation of Snail [22–25]. To determine whether CSN2 is required for the RMP-induced stabilization of Snail, the expression of CSN2 was examined by RT-PCR in HepG2 cells overexpressing RMP (RMPo). The mRNA of CSN2 was upregulated by RMP overexpression, which was similar to the effect of TNF-α (Figure 5A and Supplementary Figure 7A). Consistent with the expression of mRNA, the protein expression of CSN2 was also increased in RMPo cells treated with or without TNF-α. Surprisingly, TNF-α did not increase CSN2 without RMP overexpression (Figure 5B, lane 2 and Supplementary Figure 7B). When NF-κB activity was suppressed by BAY-11-7082, CSN2 expression was not affected by RMP as well (Figure 5B lane 6). These results indicate both RMP and NF-κB are required for the activation CSN2 expression.

To further analyze the occupation of CSN2 promoter, dual-luciferase reporter assay was carried out. The transcription of CSN2 was increased by RMP overexpression, which was further enhanced by TNF-α (Figure 5C). In contrast, the transcription of CSN2 was decreased by RMP depletion, which was further suppressed by NF-κB inhibitor (BAY-11-7082) (Figure 5C). The promoter region of CSN2 contains 3 conserved NF-κB binding sites (-1933, -1923bp), (-1283, -1273bp) and (-1198, -1188bp). We also analyzed the CSN2 promoter activity by chromatin immunoprecipitation (ChIP). CSN2 promoter was occupied by p65 in all three p65 binding sites in RMPo cells. However, p65 was absent in the CSN2 promoter when RMP was depleted compared with the HepG2 cells (Figure 5D and Supplementary Figure 7C). These results suggest that RMP activates the transcription of CSN2 through NF-κB pathway.

In order to further assess the effect of RMP and NF-κB on transcriptional activation of CSN2, cells were subjected to immunoprecipitation assay. The association of RMP and p65 was strongly enhanced in RMPo cells (Figure 5E and Supplementary Figure 8A). Moreover, when endogenous p65 was pulled down, RMP was immunoprecipitated in RMPo cell as well (Figure 5F and Supplementary Figure 8A). However, we observed weak association of RMP and p65 in HepG2 cells or RMPi cells (Figure 5E and 5F).

To further confirm the association of RMP and p65 on the activation and translocation of NF-κB, cell lysates were separated into cytoplasmic and nuclear lysates. The phosphorylation (lane 2 and 5) and nuclear localization of p65 (lane 8 and 11) were both significantly enhanced by RMP overexpression or TNF-α treatment (Figure 5G and Supplementary Figure 8B). Surprisingly, the nuclear transportation of p65 was failed in the promotion by TNF-α in RMPi cells, which is also consistent with transwell assay (Figure 4B), suggesting the correlation of RMP and p65 is required for CSN2 transcriptional activation.

### RMP activates the metastasis of HCC cells through TNF-α/NF-κB pathway *in vivo*

**Figure 6:**
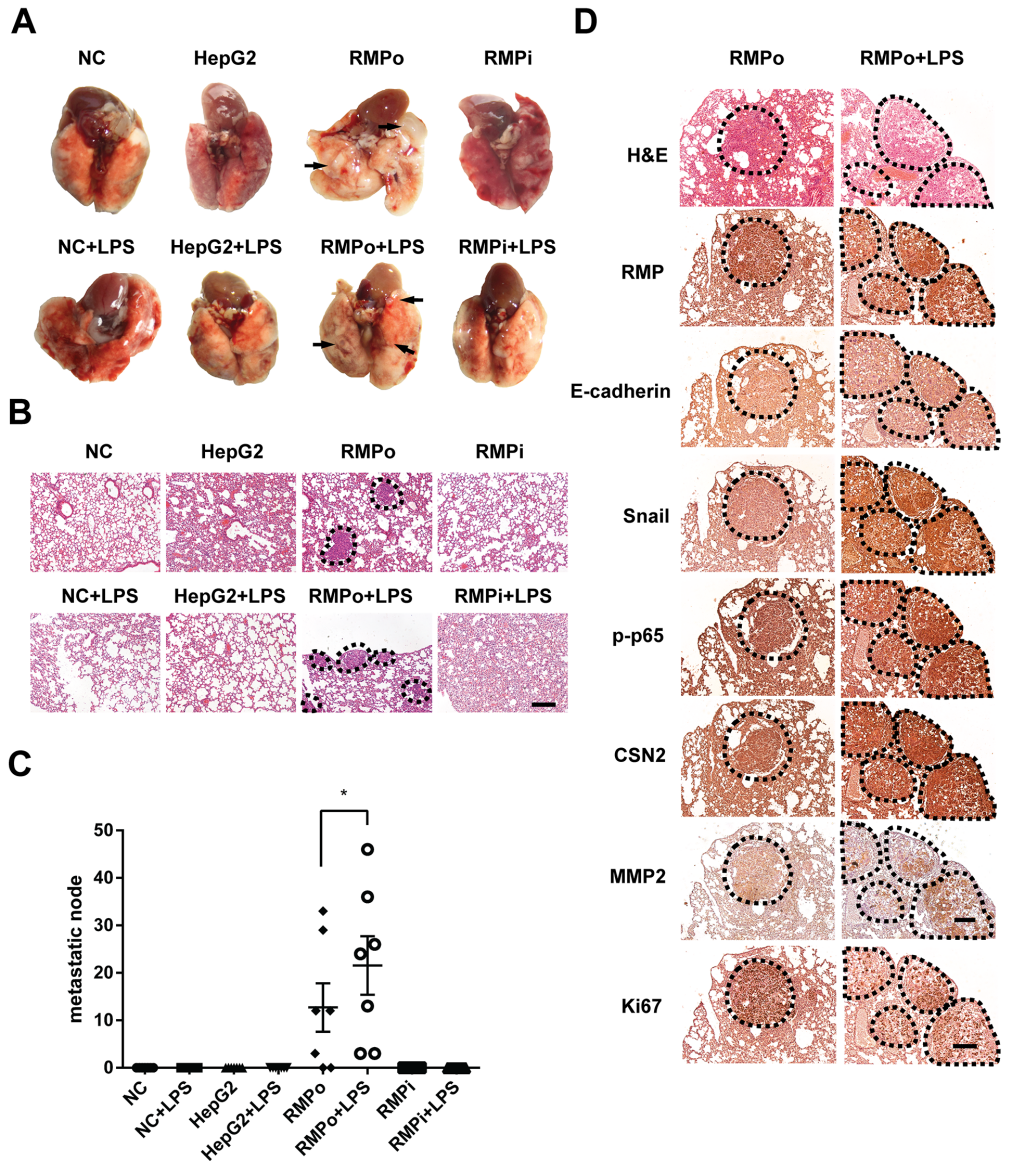
RMP activates the metastasis of HCC cells through TNF-α/NF-κB pathway *in vivo* **(A)** The lung metastasis mouse model in mice was constructed by using HepG2 cells transfected with pCDNA3.1-RMP overexpression vector (RMPo) or pGPU6-RMP shRNA vector (RMPi), n=10 per group. 5 mice in each group were additionally tail vein injected with 10 μg LPS after 14 days tumor formation. Lungs of mice were dissected at 28^th^ day. The metastatic nodules from each group are marked by arrow, Scale bar, 1 cm. **(B)** lungs of mice were sectioned and subjected into H&E staining. Dashed line marked the metastasis nodule, Scale bar 20 μm. **(C)** The lung metastases in each slide which shown in (B) was plotted and depicted graphically. Seven slides of each group was taken into account. **(D)** Tumor tissues of lung metastasis derived from RMPo cells were subjected with immunohistochemistry staining with the antibodies as indicated. Dashed line marked the metastasis nodule in serial sections. Scale bar, 20 μm. The results are expressed as the mean ±SD of three independent experiments (*, P < 0.05, **, P < 0.01, ***, P < 0.001, independent Student t test).

To investigate the effect of RMP on EMT *in vivo*, metastasis and colonization assays were carried out. HCC cells were administrated into SCID-beige mice by tail intravenous injection to generate pulmonary metastases. Moreover, half of the mice were further injected lipopolysaccharide (LPS) to activate NF-κB [26]. Surprisingly, lung metastatic nodules were only observed in mice injected with RMPo cells, which was further enhanced by LPS (Figure 6A). These lung tissues were examined by H&E staining (Figure 6B). The metastatic cells were also only observed in mice injected with RMPo cells, and RMPo cell + LPS, which was consistent with the results in (Figure 6A). Moreover, there were considerable metastatic nodules per slide in the mice injected with RMPo or RMPo+LPS, respectively (Figure 6C). However, no metastasis was observed in mice injected with HepG2 and RMPi cells (Figure 6A and 6B). To confirm the RMP-driven metastasis, immunohistochemistry analysis was carried out. Apparently, CSN2 and Snail were highly expressed in the nodules developed by RMPo cells, which was enhanced by LPS treatment (Figure 6D). Phosphorylated p65 was significantly increased by LPS in RMPo nodules. Interestingly, no typical cell membrane-located E-cadherin was observed in either metastatic nodules of RMPo or RMPo further treated with LPS (Figure 6D), indicating lung metastasis is generated mostly by mesenchymal-like cells. The expression of MMP2 was also increased in the mice injected with RMPo cells, which was further enhanced by LPS. Interestingly, Ki67, a marker of proliferation, was decreased by LPS treatment, compared with metastatic nodules without LPS treatment (Figure 6D).

### RMP regulates the expression of EMT factors in human HCC tissues

**Figure 7:**
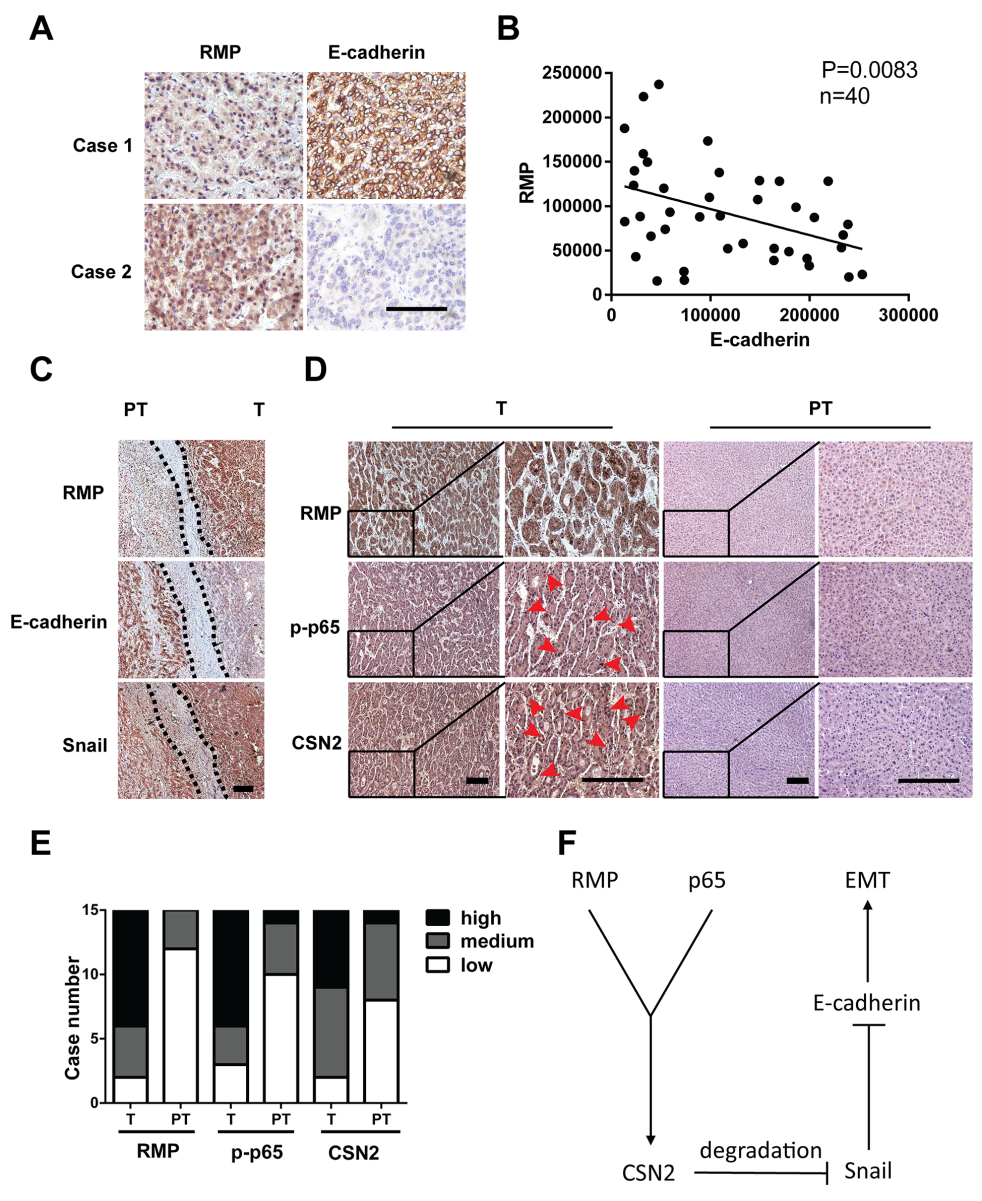
RMP regulates the expression of EMT factors in human HCC tissues **(A)** The expression of RMP and E-cadherin in human HCC tissues was determined by immunohistochemistry. Scale bar, 20 μm. **(B)** Linear regression analysis of the expression of RMP and E-cadherin in 40 human HCC tissues, showing inverse correlation between RMP and E-cadherin, P = 0.0083. **(C)** The expression of RMP, E-cadherin and Snail in invasive front of human HCC tissue was determined by immunohistochemistry, dashed line indicate the border between tumor (T) and para-tumor (PT). Scale bar, 20 μm. **(D)** The expression of RMP, p-p65, CSN2 in tumor (T) and para-tumor (PT) tissues of 15 HCC patients was determined by immunohistochemistry. Scale bar, 20 μm. **(E)** The expression of RMP, p-p65, CSN2 as determined in (D) was measured and calculated. The expression of these factors was classified into high, medium and low. **(F)** Model proposing a role of RMP and p65 induced stabilization of Snail in EMT.

To further investigate the clinical significance of RMP in EMT of HCC, we examined the expression of RMP and EMT factors in 40 HCC patients by immunohistochemical (IHC) analysis, The results showed that increased expression of RMP was always correlated with the decrease of E-cadherin (Figure 7A), reverse correlation were observed between RMP and the epithelial marker E-cadherin (Figure 7B). RMP expression in tumor tissues (T) was stronger than that in its corresponding para-tumor tissues (PT), indicating RMP is an inhibitor of E-cadherin. More importantly, a positive correlation was observed between RMP and Snail, which was highly expressed in the metastatic front of HCC (Figure 7C).

Next, we examined the correlation between RMP, p65 and CSN2 in human HCC tissues. As shown in (Figure 7D), the nuclear localization of p65 (12 of 15 cases) and CSN2 (13 of 15 cases) in HCC tumor tissues (T) (red arrow) was higher than their para-tumor tissues (PT), which is highly consistent with the enhanced expression of RMP in these tissues (Figure 7D and 7E). Collectively, RMP was found to be significantly associated with activation of p65, CSN2 and Snail, which were involved in the invasion and metastasis of HCC.

## DISCUSSION

Recent studies uncovered more and more evidences of that RMP promoted carcinogenesis. RMP expression was found to increase in liver cancer, ovarian cancer and portal vein tumor thrombus (PVTT), which was responsible for series of malignant phenotype of these cancer [7, 8, 12]. In this study, we found that RMP promotes the migration, invasion and EMT of HCC cells *in vitro* and HCC metastasis *in vivo*. Remarkably, our study demonstrates a mechanism of stabilizing Snail by NF-κB/CSN2 axis in the process of RMP-induced EMT and cancer metastasis.

Snail is a critical transcription factor involved in EMT initiation in human cancers [4]. The nuclear export and cytosolic degradation of Snail is mostly charged by PI3K/Akt downstream factor GSK-3β, and activation of Akt is able to increase the stabilization of Snail by inhibiting GSK-3β activity [27]. However, in our studies, we found instead of Akt, the activation of NF-κB pathway increased the stability of Snail in RMP-induced EMT in HCC. Similar to our findings, RMP/URI knockdown did not affect the phosphorylation of Akt/PKB and its downstream factor 4E-BP1 in Hela cells during serum starved or IGF treatment [28].

CSN2 is an essential component of COP9 signalosome complex (CSNs), which is the regulator of the ubiquitin conjugation pathway. CSNs is also involved in phosphorylation of IκB/NF-κB, p53/TP53, c-jun/JUN and response for their protein stability by regulated through ubiquitylation and degradation [29]. In this study, we found RMP up-regulated CSN2 expression through promoting nuclear translocation of NF-κB. The interaction between RMP and p65 may release p65 from its inhibitor IκB, promoting the phosphorylation and nucleus localization of p65. Since the promoter of CSN2 contains at least 3 p65 binding sites, the CSN2 transcription is activated by the nuclear p65. Thereafter, CSN2 may block the phosphorylation and ubiquitylation of Snail by disrupting its binding to GSK-3β and β-TRCP [26], which in turn triggered EMT in HCC. Consistent with our hypothesis, blocking p65 activity inhibited the transcription of CSN2, disrupted Snail stabilization and attenuated the invasive capability of the cells regardless of high RMP level.

Furthermore, Snail was found to be involved in an anti-apoptotic function in addition to the induction of EMT [30, 31]. Promoting Snail degradation also induces apoptosis and thus contributes to the metastatic suppression [32], which can partially explain why no lung metastasis was found in mice injected with wild type HepG2 cells or cells depleted of RMP.

In summary, we investigated the function of RMP in promoting the migration, invasion, EMT of HCC cells and HCC metastasis. We further demonstrated the role of NF-κB/CSN2/Snail axis in RMP-mediated EMT. A comprehensive understanding of the role of RMP in HCC will speed up the discovery of strategies for HCC therapy.

## MATERIALS AND METHODS

### Plasmids, siRNA, and antibodies

RMP shRNA expression plasmid pGUP6-RMPi and overexpression plasmid pCDNA3.1-RMPo was purchased from Gene Pharma (Shanghai, China). Antibodies of RMP, Ser-473 phosphorylated AKT, Ser-9 phosphorylated GSK-3β, GSK-3β, Snail, E-cadherin, Ser-32 phosphorylated iκB, iκB and lamin B was purchased from Santa Cruz Biotechnology (Dallas, USA); Antibody of p65 was purchased from Abcam (Cambridge, UK); Antibody of CSN2 was purchased from Proteintech (Chicago, USA); Ser-536 phosphorylated p65 was purchased from Cell Signaling Technology (Danvers, USA).

### Human tumor samples and immunohistochemistry

Tumor specimens were obtained from The Third Affiliated Hospital of Soochow University, China, and approved by the ethics committee. All 40 pairs of HCC patient specimen were recruited to testing for immunohistochemistry. The procedure of immunohistochemistry was performed as described previously [9].

### Sphere assay and colony formation assay

For sphere assay, DMEM semisolid medium made by 20% FBS, 2× DMEM complete medium 1:1 mixed with low melting agarose (Sigma Aldrich). HepG2, RMPo and RMPi cells (1×10^3^ each) were premixed with 2 ml semisolid DMEM medium in 37°C and plate into 6-well plate, cultured for 14 days in 37°C 5% CO_2_, images of the spheres was captured. Spheres (diameter ≥ 10μm) were counted. The colony formation assay was carried out as described previously [8].

### Wound healing assay

Cells were seeded in fibronectin coated 6-well plate, and wounds were made by 200 μl pipette tips when the cell reached 90% confluence. Detached cells were washed by PBS and then cultured in fresh serum-free DMEM medium. The photographs were taken at 0, 12 and 24 hrs. The wound width was quantified by NIS-Elements software (Nikon, Japan).

### Cell invasion and migration assay

For invasion assay, HepG2 and Huh7 cell were transfected with pCDNA3.1-RMPo or pGUP6-RMPi or their respective control for 48 hrs, 5×10^4^ cells were suspended in serum-free DMEM medium and seeded in the top chamber of 24-well transwell unit (Corning) which pre-coated with 1: 8 diluted matrigel (BD pharmingen), DMEM with 10% FBS was added to the bottom chambers to induce metastasis. Cells were allowed to migrate for 24 hrs at 37°C, and then un-invaded cells in the top chambers were removed and cells which migrated into the bottom chambers were fixed, stained and quantified. For the migration assay, the cells which indicated in invasion assay were seeded into the top chambers without coated, and followed by the steps of invasion assay.

### Zymographic assay

Supernatants of cells were prepared in non-denaturation 2×SDS sample buffer and loaded without boiling to a 10% polyacrylamide gel copolymerized with 1mg/ml of gelatin. The Zymographic assay was carried out as described previously [33].

### RT-PCR

RT-PCR was carried out as described previously [8]. The sequences of primers involved in PCR reaction were as follows: CSN2 (Forward: 5′-AAA TAT GCT TAT GAA ATC GGG AA-3′; reverse, 5′-TGA TAG GCA CTT ACT AAA TTC GT-3′), Snail (Forward: 5′-TTC TCT AGG CCC TGG CTG C-3′; reverse, 5′-TAC TTC TGA CAT CTG AGT GGG TCT G-3′), Slug (Forward: 5′-CTG GGC TGG CCA AAC ATA AG-3′; reverse, 5′-CCT TGT CAC AGT ATT TAC AGC TGA AAG-3′), Twist1 (Forward: 5′-GCA GGA CGT GTC CAG CTC-3′; reverse, 5′-CTG GCT CTT CCT CGC TGT T-3′), Twist2 (Forward: 5′-GCA AGA AGT CGA GCG AAG AT-3′; reverse, 5′-GCT CTG CAG CTC CTC GAA-3′), ZEB1 (Forward: 5′-TAC AGA ACC CAA CTT GAA CGT CAC A-3′; reverse, 5′-GAT TAC ACC CAG ACT GCG TCA CA-3′), RMP (Forward: 5′-TGT CCC TCG CAA ATC CAT CCT G-3′; reverse, 5′-CTC CTC AAA ACT CCC CGC CTA-3′), GAPDH (Forward: 5′-GAC CTG ACC TGC CGT CTA-3′; reverse, 5′-AGG AGT GGG TGT CGC TGT-3′), Each PCR products were separated in 2% agarose gel.

### ChIP assay

ChIP assay was performed according to the instruction manual of Chromatin immunoprecipitate kit (Millipore). Briefly, cells were cross link chromatin by 1% formaldehyde before cell lysis, DNA fragmentation by sonication with 3 times 10sec each. Cell lysates were then precleared using Salmon Sperm DNA/Protein A/G Agarose, and the lysates were immunoprecipitated with p65 antibody. Finally, the DNA from the immunoprecipitation was purified and subjected into PCR amplification. The primer sequences were show as follows, CSN2 promoter region 1 (forward: 5′-CAT GGG ACT TAA CTA TTT GTC A-3′; reverse, 5′-CCA AAT AAA GGC ACA TAG GAG-3′);CSN2 promoter region 2 & 3 (forward: 5′-TGA GAG CAT TAA ACA CTT GGT-3′; reverse, 5′-CGC AAG AAT ATC ATT TAG AAC AGA-3′); CSN2 CDS region (forward: 5′-TGA AGA TAG TAA CTC CGA GCC AA-3′; reverse, 5′-CTC CGA ATA TAG GTC AAT AGC TG-3′).

### Dual-luciferase reporter assay

Cells of 70% confluence in 96-well plates were transfected using lipofectamine 3000 (life technology). The pGL3-promoter-CNS2 firefly luciferase reporter gene construct (100ng) and the pGL4.74 Renilla luciferase control vector (10ng) were used for co-transfection. The cells were subject to dual-luciferase assay 24hrs after transfection, and the luciferase activity was measured using the Dual-Luciferase Reporter Assay System (Promega).

### Experimental lung metastasis model

Female SCID-beige mice (6-8 weeks old) were purchased from animal experiment center of Soochow University and maintained and treated under specific pathogen-free conditions. All animal protocols were approved by the Institutional Laboratory Animal Care and Use Committee at Soochow University. Mice (20 for each group) were injected intravenously with 2×10^6^ of HepG2, RMPo, RMPi cells and PBS via the tail vein respectively. Four weeks later, half mice (10) of each group were injected intraperitoneally with 10 μg of LPS (055: B5; Sigma) in normal saline or with normal saline alone. After visible lung metastatic nodules were examined, the lungs were followed by paraffin-embedded section.

## SUPPLEMENTARY MATERIALS FIGURES


