# Supplementary material for: RMP promotes epithelial-mesenchymal transition through NF-κB/CSN2/Snail pathway in hepatocellular carcinoma
Source: Oncotarget. 2017 Mar 14;8(25):40373–88. doi: 10.18632/oncotarget.16177 (PMC5522250; doi:10.18632/oncotarget.16177)
Supplement: Supplementary file 1 [file oncotarget-08-40373-s001.pdf]

RMP promotes the epithelial-mesenchymal transition through NF-κB/CSN2/Snail pathway in hepatocellular carcinoma

SUPPLEMENTARY MATERIALS

Quantitative RT-PCR

Quantitative RT-PCR was performed to measure RMP, CD44, CD90, CD133, EpCAM, Snail, Slug, Twist1, Twist2, ZEB1 mRNA levels. Total RNA was isolated from cells using TRIzol reagent according to the manufacturer's instructions. Glyceraldehyde 3-phosphate dehydrogenase (GAPDH) gene expression served as an internal control. Reactions were performed using thermal cycle as described previously (Yang HC, etc. Journal Of Biological Chemistry. 2011; 286(13):11865-11874). The Ct value of each gene was determined after normalization to GAPDH, and $^{\Delta\Delta}$Ct was calculated relative to the designated reference sample. The fold-change in gene expression was calculated from $2^{\Delta\Delta Ct}$.

Densitometric analysis

The Gray scale density was taken by Photoshop software, and normalized by internal control with β-actin or Lamin B as indicated.

SUPPLEMENTARY FIGURES

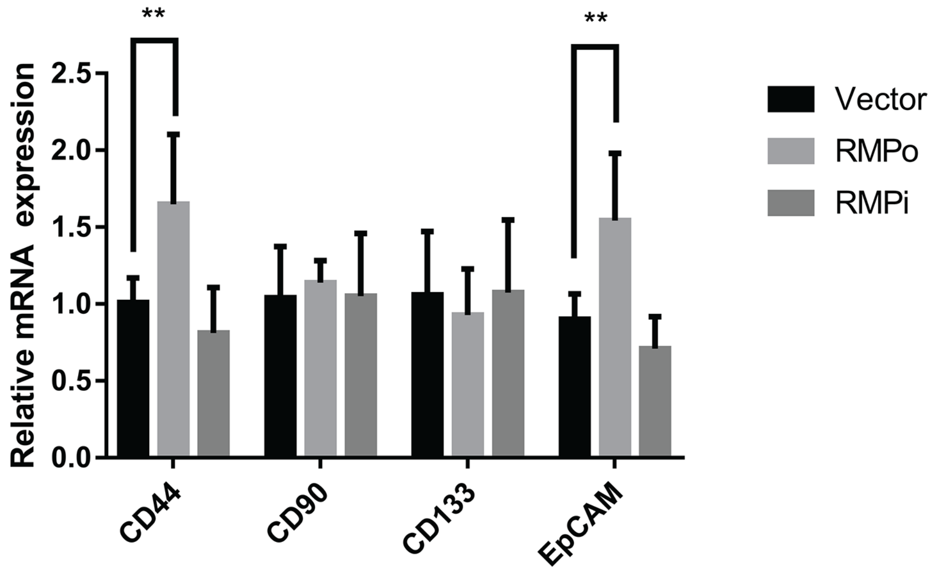

Supplementary Figure 1: The expression of CD44, CD90, CD133 and EpCAM was analyzed by QPCR in HepG2 cells transfected with vector, RMPo or RMPi, respectively. The results were expressed as the mean ±SD of three independent experiments, each measurement was made in triplicates (*, P< 0.05, **, P < 0.01, ***, P < 0.001, ****, P < 0.0001, two-way ANOVA).

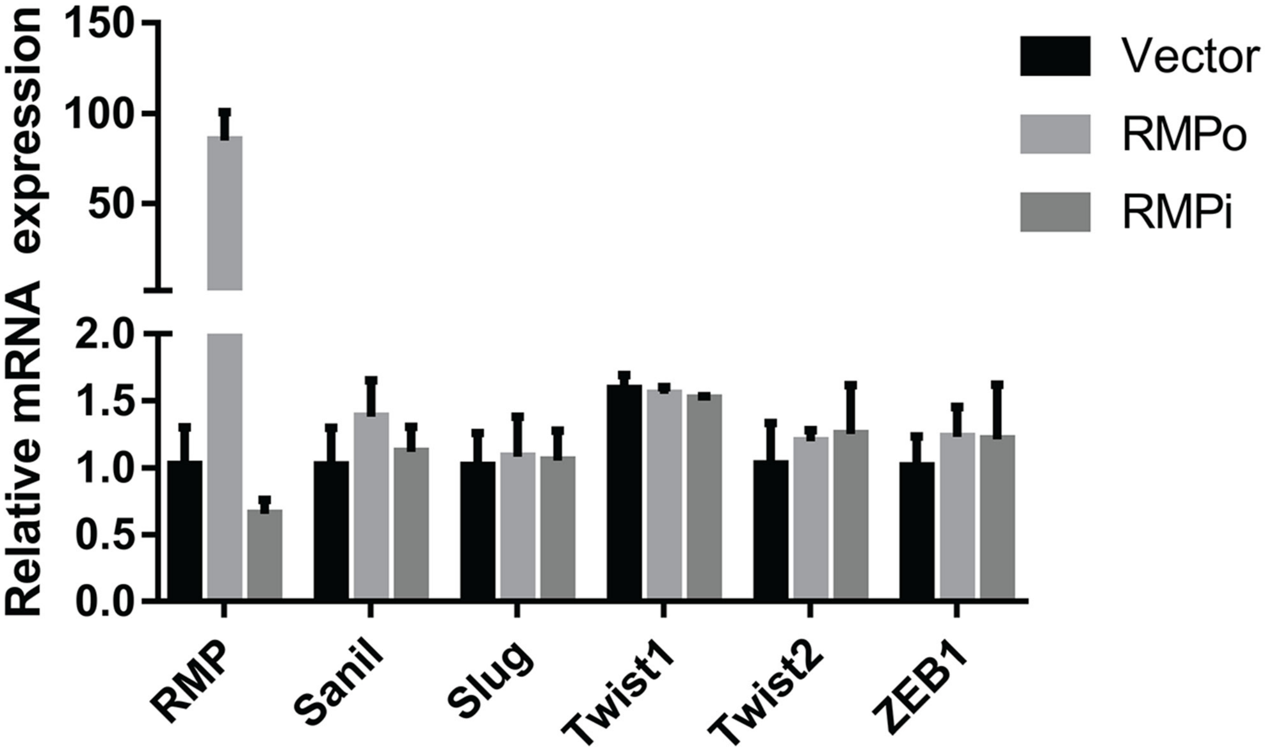

Supplementary Figure 2: The expression of indicated genes were analyzed by QPCR in HepG2 cells transfected with vector, RMPo or RMPi, respectively. The results were expressed as the mean ±SD of three independent experiments, each measurement was made in triplicates (*, $P< 0.05$, **, $P< 0.01$, ***, $P < 0.001$, ****, $P < 0.0001$, two-way ANOVA).

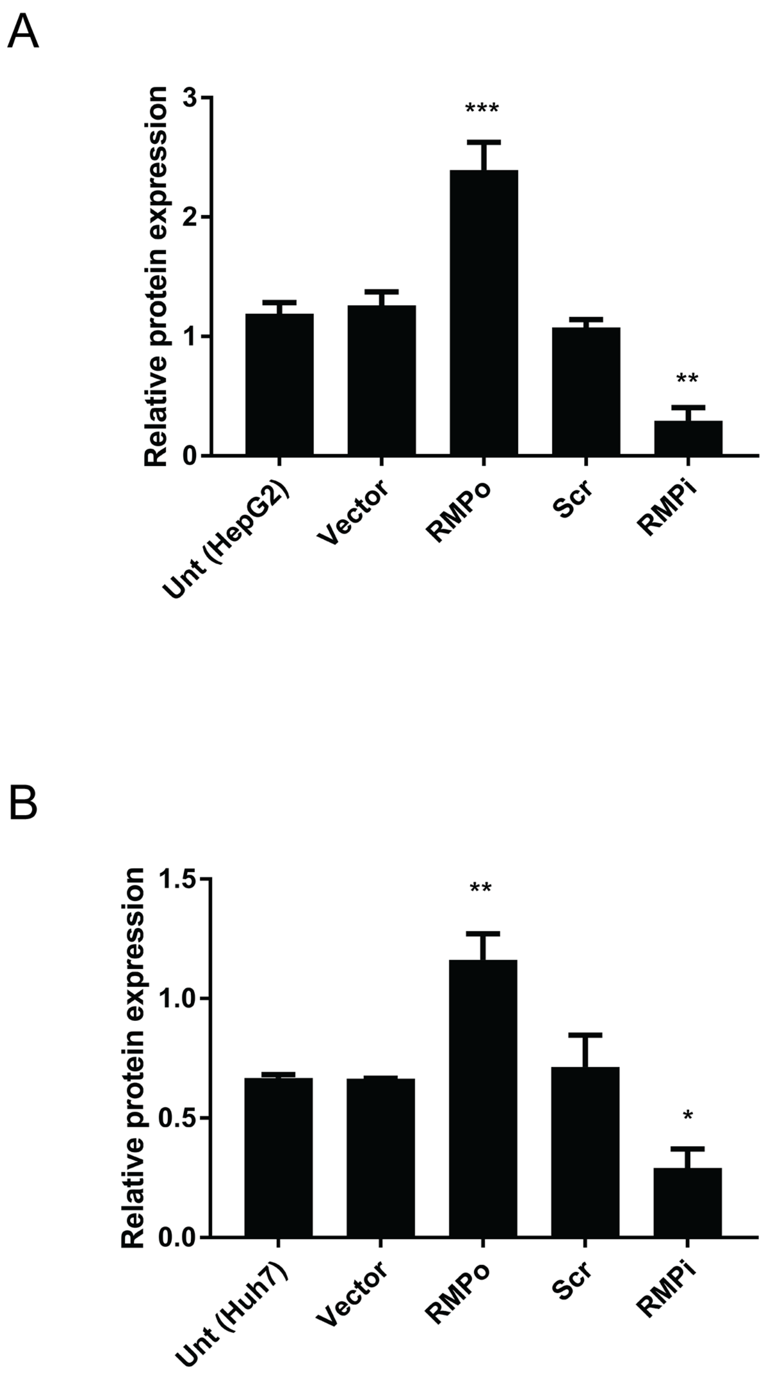

Supplementary Figure 3: Densitometric analysis for the western blot in Figure 1A showing RMP expression in HepG2 **(A)** or Huh7 cells **(B)** transfected with indicated vectors. The results were expressed as the mean ±SD of three independent experiments, each measurement was made in triplicates (*, P< 0.05, **, P < 0.01, ***, P < 0.001, ****, P < 0.0001, Ordinary one-way ANOVA).

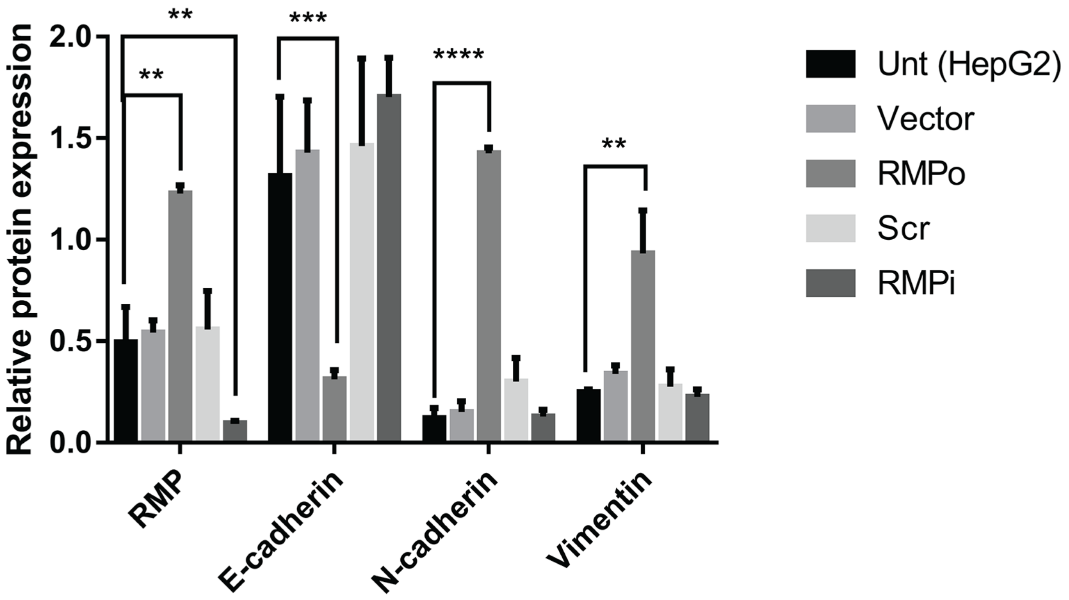

Supplementary Figure 4: Densitometric analysis for the western blot in Figure 2E showing the expression of indicated genes in cells transfected with various vectors. The results were expressed as the mean ±SD of three independent experiments, each measurement was made in triplicates (*, P < 0.05, **, P < 0.01, ***, P < 0.001, ****, P < 0.0001, two-way ANOVA).

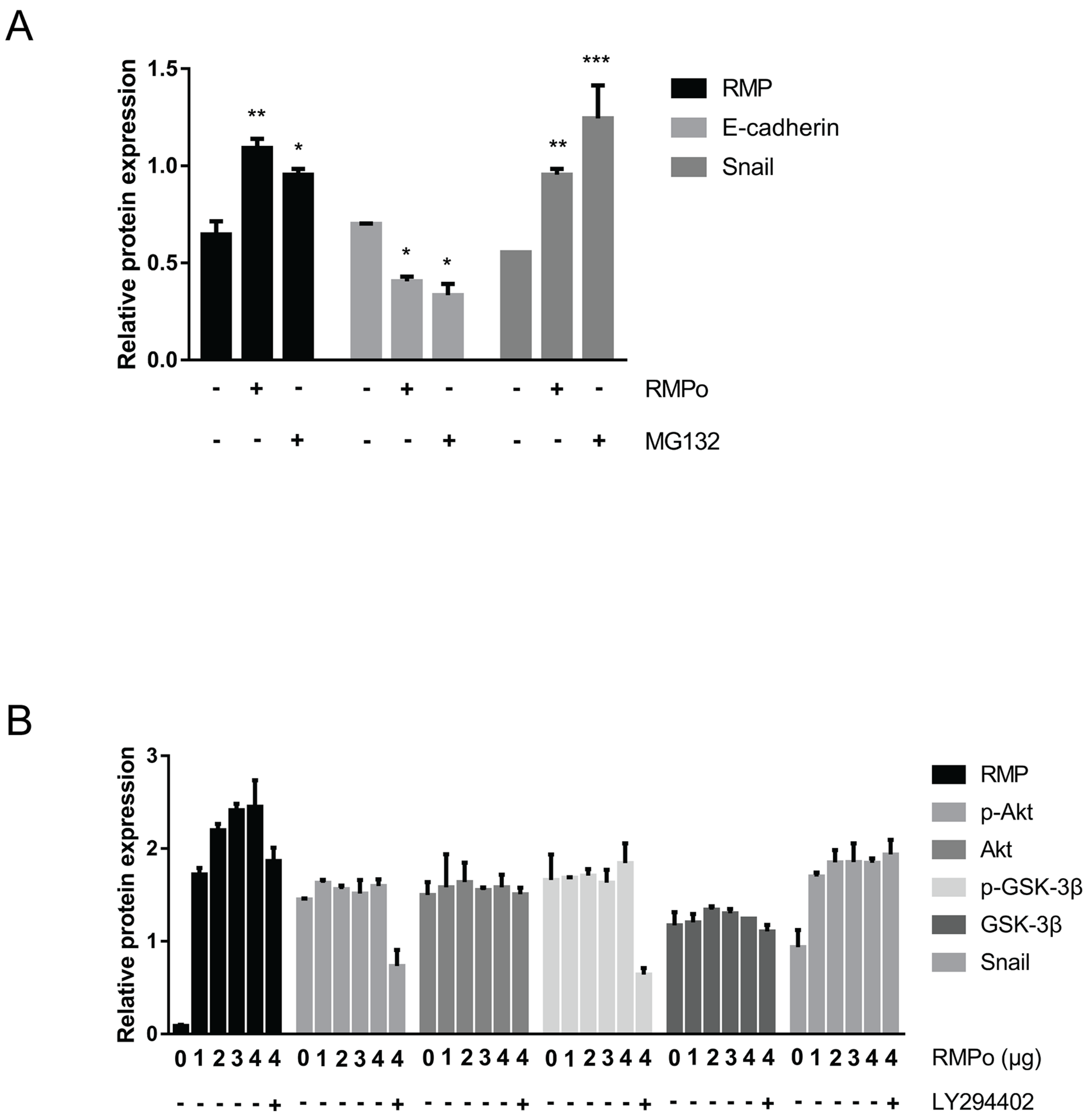

Supplementary Figure 5: (A) Densitometric analysis for the western blot in Figure 3B showing the protein expression of RMP, E-cadherin and Snail in HepG2 cells transfected with RMPo or treated with MG132. **(B)** Densitometric analysis for the western blot in Figure 3C showing the expression of various proteins when cells were transfected with RMPo in a dose dependent manner. These results were expressed as the mean ±SD of three independent experiments, each measurement was made in triplicates (*, $P < 0.05$, **, $P < 0.01$, ***, $P < 0.001$, ****, $P < 0.0001$, two-way ANOVA).

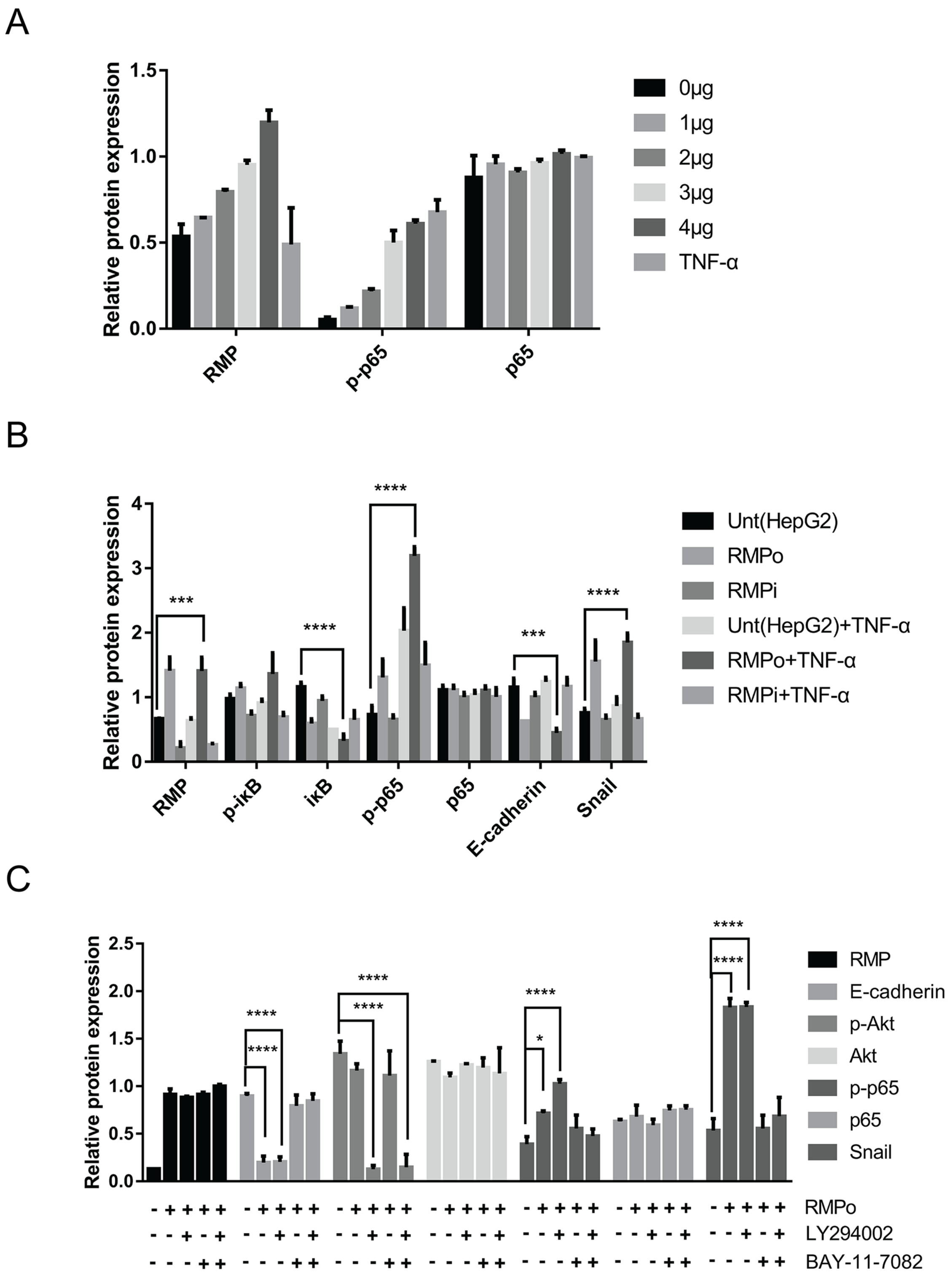

Supplementary Figure 6: (A) Densitometric analysis for the western blot in Figure 4A showing the expression of RMP, p-p65 and p65 when cells were transfected with RMPo in a dose dependent manner. **(B)** Densitometric analysis for the western blot in Figure 4C showing the expression of various proteins in HepG2, RMPo and RMPi cells with or without TNF-α treatment. **(C)** Densitical analysis for the western blot in Figure 4D showing expression of various genes in HepG2 cells with treatments as indicated: transfection with RMPo vector, treatment with LY294002 or BAY-11-7082. These results were expressed as the mean ±SD of three independent experiments, each measurement was made in triplicates (*, $P < 0.05$, **, $P < 0.01$, ***, $P < 0.001$, ****, $P < 0.0001$, two-way ANOVA).

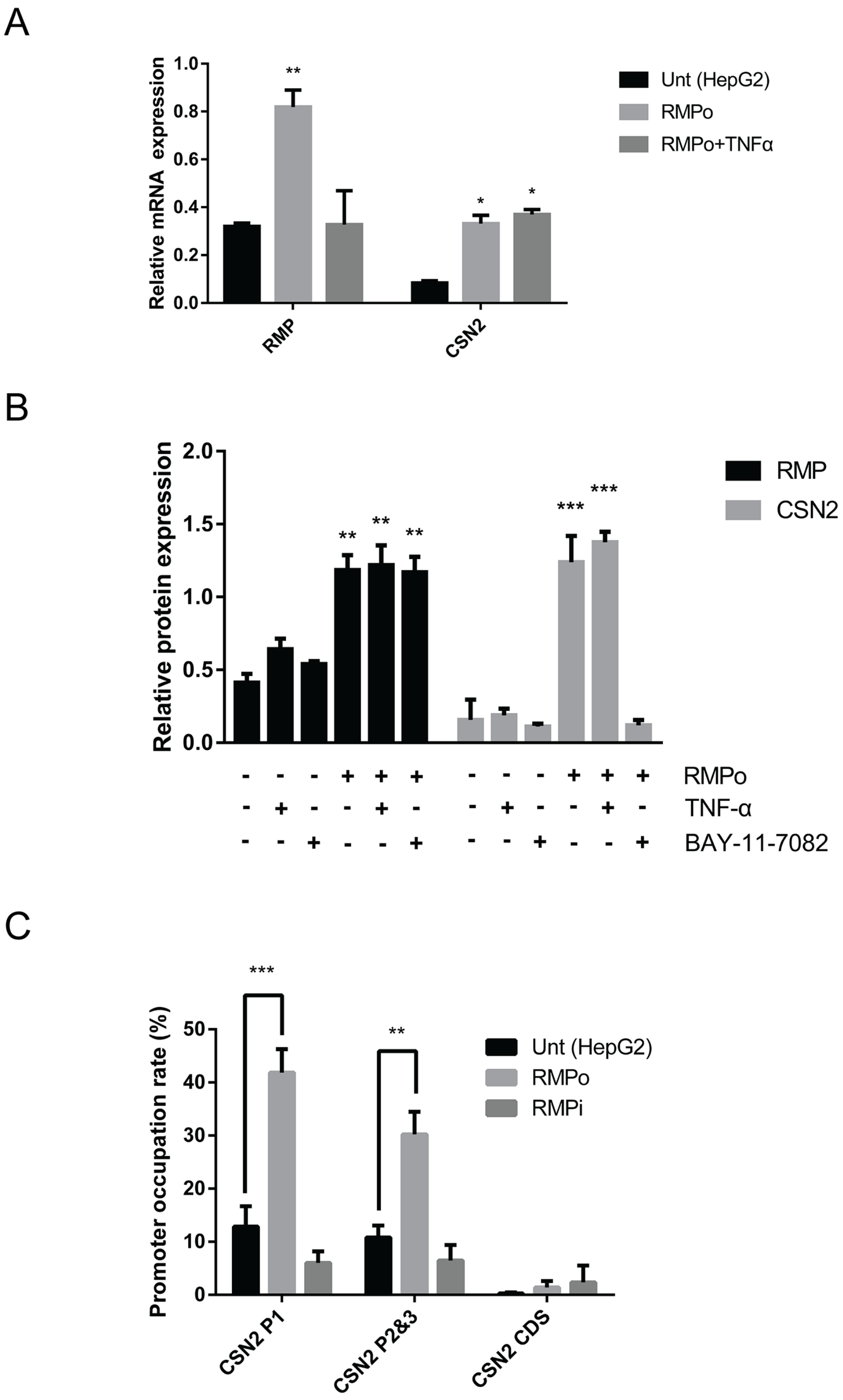

Supplementary Figure 7: (A) Densitometric analysis for the RT-PCR in Figure 5A showing the expression of RMP and CSN2 during HepG2 cells transfected with RMPo vector with or without TNF-α treatment. **(B)** Densitometric analysis for the western blot in Figure 5B showing the expression of RMP and CSN2 in HepG2 cells with treatments as indicated: transfection with RMPo vector, treatment with TNF-α and BAY-11-7082. **(C)** Densitometric analysis for ChIP assay in Figure 5D, showing the ratio of promoter occupation by p65. These results were expressed as the mean ±SD of three independent experiments, each measurement was made in triplicates (*, $P < 0.05$, **, $P < 0.01$, ***, $P < 0.001$, ****, $P < 0.0001$, two-way ANOVA).

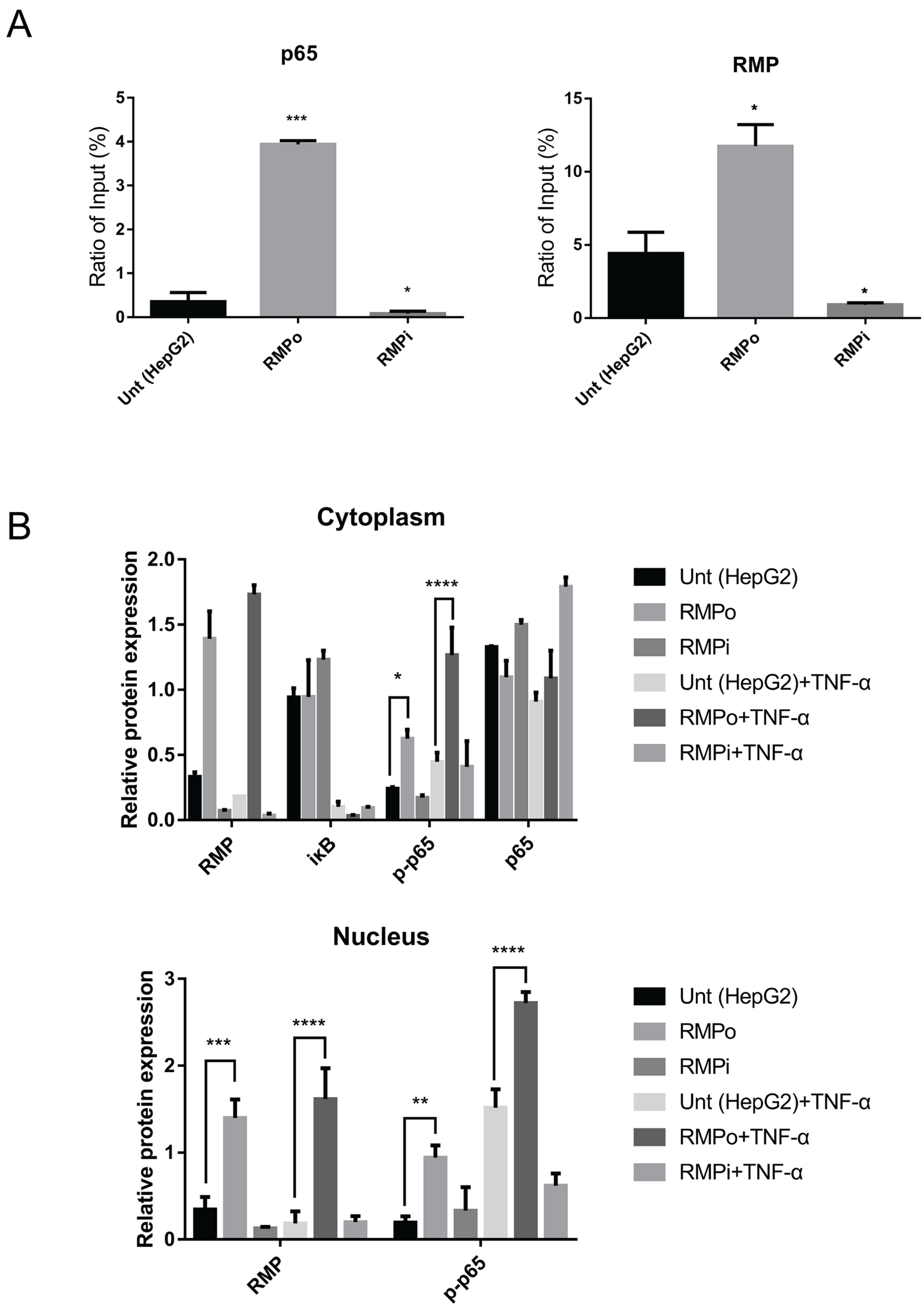

Supplementary Figure 8: (A) The fold change of immunoprecipitation assay in Figure 5E and F. **(B)** Densitometric analysis for the western blot in Figure 5G showing the expression of indicated genes in the cytoplasm and nucleus in cells transfected with various vectors. These results were expressed as the mean ±SD of three independent experiments, each measurement was made in triplicates (*, $P < 0.05$, **, $P < 0.01$, ***, $P < 0.001$, ****, $P < 0.0001$, two-way ANOVA).
